# Dental implants in Sjögren’s syndrome patients: A systematic review

**DOI:** 10.1371/journal.pone.0189507

**Published:** 2017-12-14

**Authors:** Daniel Almeida, Katia Vianna, Patrícia Arriaga, Vittorio Moraschini

**Affiliations:** 1 Brazilian Air Force, Odontoclínica de Aeronáutica Santos-Dumont, Department of Implantology, Centro, Rio de Janeiro, Brazil; 2 Fluminense Federal University - Department of Periodontology - School of Dentistry, Centro, Niterói, Rio de Janeiro, Brazil; Virginia Commonwealth University, UNITED STATES

## Abstract

**Objectives:**

The Sjögren’s syndrome (SS) is a chronic autoimmune disease that affects salivation and consequently the health of oral tissues. The aim of this systematic review was to investigate the implant survival rate, marginal bone loss (MBL) and biological complications of dental implants in SS patients.

**Materials and methods:**

Eligibility criteria included prospective and retrospective cohort studies, controlled clinical trials, and randomized clinical trials (RCTs). An electronic search without date or language restrictions was carried out in MEDLINE, Cochrane, Web of Science, and LILACS until June 2017. In addition, manual search and in the grey literature were also conducted. The search process, data analysis, and quality assessment were performed by two independent reviewing authors. The protocol of this systematic review was registered in PROSPERO under number CRD42016053277.

**Results:**

The search and selection process yielded 6 studies, published between 1997 and 2016. An average of 93.7% survival in a mean period of 3.97 years of follow-up was observed. A low number of MBL and biological complications were reported by the studies. All the studies analyzed observed an improvement in life quality of subjects with SS and rehabilitated through dental implants.

**Conclusions:**

With the limitations of this review and based on the available data, the dental implant therapy in SS patients seems to present high implant survival rate, low MBL and low biological complications. In addition, all included studies observed an increase in the quality of life of SS patients who were rehabilitated through dental implants.

## Introduction

The use of dental implants is safe and predictable [1]. However, some local or systemic conditions have been associated to dental implant failures such as low insertion torque, peri-implant disease, smoking, bruxism, diabetes, and bisphosphonates [2–6].

The SS is an autoimmune rheumatic disease characterized by focal mononuclear cell infiltration of the salivary and lachrymal glands [7]. SS has been suggested to affect 0.2% to 3.0% of the population [8–10]. It predominantly affects women between 40 and 60 years of age, with a 9:1 female/male ratio. Younger individuals and children may also be affected [11].

Primary SS occurs solitarily, whereas secondary SS occurs in association with other autoimmune diseases, most frequent being rheumatoid arthritis and systemic lupus erythematosus [12].

The most common and earliest symptoms of SS are oral and ocular dryness. Dry mouth leads to difficulty in talking, tasting, and chewing properly, impairing quality of life of these patients. The most common oral signs and symptoms are hyposialia with or without xerostomia, tooth decay, fungal infections, traumatic oral lesions, dysphagia, dysgeusia, and inflammation of salivary glands [13]. The resulting xerostomia increases the development of dental plaque and the likelihood of periodontal disease [14].

The oral consequences of this pathologic process are: higher number of decayed, missing and filled teeth; and higher plaque index, gingival index and papillary bleeding index when patients with and without SS are compared [15]. Thus, it is very common that these patients require dental implants to rehabilitate any extractions arising from decay or periodontal disease.

In addition, such patients are often treated with immunomodulators (e.g., hydroxychloroquine, methotrexate) and sometimes with immunosuppressive drugs, reducing and changing the patient’s immune response [16]. As of the date of this work the authors found no systematic review to assess the survival or success rates of implants placed in patients with SS.

The aim of this systematic review was to investigate the implant survival rate, marginal bone loss and biological complications of dental implants in SS patients.

## Materials and methods

The protocol of this review was based primarily on the PRISMA-P [17] and registered in PROSPERO under number CRD42016053277. This SR’s methodology followed the recommendations of the Cochrane Handbook for Systematic Reviews of Interventions [18]. PRISMA [19] (S1 Fig) guidelines and AMSTAR [20] checklists were followed in order to increase the quality and transparency of the search. Clinical questionnaires were separated and organized using the PICOS strategy [21].

### Focused question

What are the clinical outcomes of implants placed in patients with SS?

### Clinical relevance

The SS is a chronic autoimmune disease that affects salivation and consequently the health of oral tissues. The clinic results of this review provide scientific evidence about the impact of SS on the predictability of dental implants.

### Outcome measures

The primary outcome was to verify implant survival. Secondary outcomes were to evaluate the level of MBL, incidence of biological complications, and improvement in life quality (masticatory function, comfort, and satisfaction) of subjects with SS after treatment through dental implants.

### Search strategy

An electronic search without restriction of dates or language was conducted on PubMed/MEDLINE, Cochrane Central Register of Controlled Trials, Web of Science and EMBASE until June of 2017. In addition, a specific electronic search in the following journals was also conducted: *Journal of Periodontology*, *Journal of Clinical Periodontology*, *International Journal of Periodontics & Restorative Dentistry*, *Clinical Oral Implants Research*, *Clinical Implant Dentistry and Related Research*, *The International Journal of Oral & Maxillofacial Implants*, *International Journal of Oral & Maxillofacial Surgery* and *Implant Dentistry*. A search for unpublished studies (grey literature) was conducted on Grey Literature Report and OpenGrey databases. Searches in the ClinicalTrials.gov database and in the references of the included studies (cross referencing), were also conducted.

MeSH terms, keywords, and other free terms related to “Sjögren syndrome[MeSH]”, Sicca syndrome[MeSH], xerostomia[MeSH], hyposialia[All Fields], Dental implant[MeSH], dental implant surgery[All Fields], dental implantation[MeSH], dental implant rehabilitation[All Fields] were used with Boolean operators (OR, AND) to combine searches. The same keywords were used for all search platforms followed the syntax rules of each database. The search strategy and PICOS tool are presented in Table 1.

**Table 1:** Systematic search strategy (PICOS strategy).

**Search strategy**	
**Focused question**	What are the clinical outcomes of implants placed in patients with SS?
**Search strategy**	
Population	#1. (Partially edentulous OR edentulous jaw[MeSH] OR edentulous maxilla OR edentulous mandible OR Sjögren syndrome[MeSH] OR Sjögren s syndrome[MeSH] OR Sicca syndrome[MeSH] OR xerostomia[MeSH] OR hyposialia)
Intervention	#2. (Dental implant[MeSH] OR dental implant surgery OR dental implantation[MeSH] OR single implant OR multiple implant OR dental implant rehabilitation)
Comparisons	#3. Not applicable
Outcomes	#4. (Cumulative survival rate[MeSH] OR survival OR dental implant survival OR dental implant failure OR failure OR marginal bone loss OR implant bone resorption OR dental implant bone loss)
Study design	Prospective cohort, retrospective cohort, case series, controlled clinical trial, and randomized controlled trial
Search combination	#1 AND #2 AND #3 AND #4
**Database search**	
Language	No restriction
Eletronic databases	PubMed/MEDLINE, Cochrane Central Register of Controlled Trials, Web of Science and EMBASE
Journals	*Journal of Periodontology*, *Journal of Clinical Periodontology*, *Clinical Oral Implants Research*, *Clinical Implant Dentistry and Related Research*, *The International Journal of Oral & Maxillofacial Implants*, *International Journal of Oral & Maxillofacial Surgery*, and *Implant Dentistry*.
Grey literature	Grey Literature Report and OpenGrey

### Inclusion criteria outlines according to the population, interventions, comparisons, outcomes, and study design (PICOS strategy)

Population (P): adult volunteers (≥ 18 years) with SS and rehabilitated through dental implants.

Interventions (I): rehabilitation of partial or total edentulous through dental implants.

Comparison (C): dental implants outcomes in participants with or without SS.

Outcome (O): dental implant survival, MBL, biological complications, and quality of life.

Study design (S): Prospective cohort, retrospective cohort, case series, controlled clinical trial, and (RCTs).

### Exclusion criteria

Animal studies, *in vitro* studies, case reports, and reviews. In addition, studies in volunteers with periodontal disease without prior treatment and studies that included participants aged <18 years, were excluded.

### Selection criteria

This review included prospective or retrospective cohort studies, case series, controlled clinical trial, and randomized controlled trial that evaluated total or partial edentulous patients with with SS (>18 years) rehabilitated through dental implants. Animal studies, *in vitro* studies, case reports, and reviews were excluded. Studies in volunteers with periodontal disease without prior treatment and studies that included participants with others metabolic diseases, were also excluded.

### Screening process

The search and screening process was carried out by two independent reviewing authors (V.M.F. and D.A.), following the previously established eligibility criteria, first analyzing titles and abstracts. In a second phase, complete articles were selected for careful reading and analyzed per eligibility criteria (inclusion/exclusion) for future data extraction. Discrepancies among authors/reviewers were resolved through careful discussion. The search agreement between the two reviewers was evaluated by the Cohen’s Kappa (k) test. If needed, the authors of the included studies were contacted by e-mail for clarification of any doubts.

### Data extraction

The following data were extracted from the included studies (when available) by two independent reviewing authors (V.M. and D.A.): authors, study design, follow-up, number of subjects, age, gender, Sjögren type, mean survival rate, implant brand, implant size, study enviroment, number of smokers, marginal bone loss, and author’s conclusions.

### Assessments of the risk of bias and quality

Risk of bias and study quality analyses were performed independently by two reviewing/authors (V.M. and K.V.). For the analysis of non-randomized studies (prospective and retrospective cohort studies and case series), the Newcastle-Ottawa scale (NOS) (http://www.ohri.ca/programs/clinical_epidemiology/oxford.asp) was used. For the selection categories and result, the studies can get a star/point for each item. For the comparison category, two stars/points can be assigned. According to NOS, the maximum score assigned to a study is nine stars/ points. Studies rated 6 stars and up are considered as high quality.

### Statistical analysis

The mean implant survival and follow-up period were calculated by summing the values reported by the studies and dividing by the total number of events.

For descriptive statistics, the Excel program (Mac. 2015, version 15.13.3, Microsoft) was used.

## Results

### Literature search

**Fig 1:**
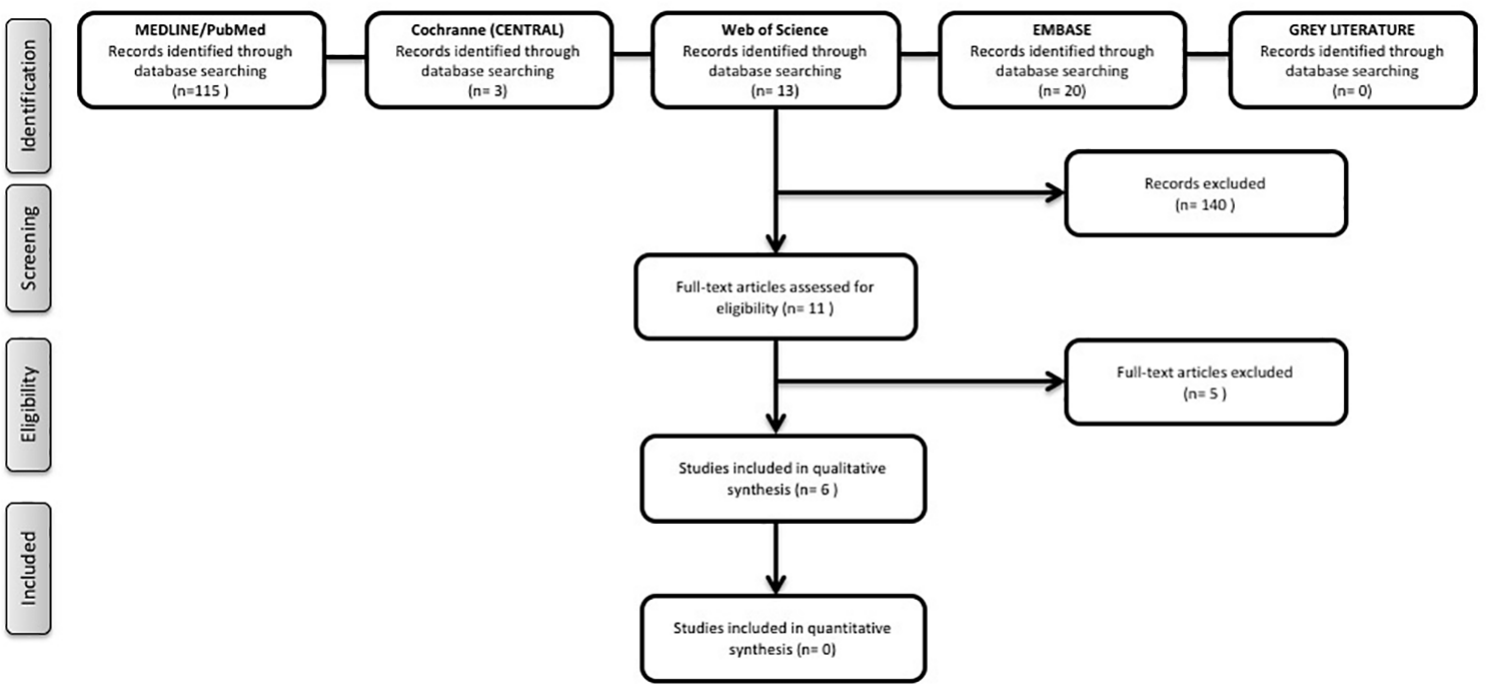
“PRISMA flow diagram of the screening and selection process”.

The initial search resulted in 115 titles in MEDLINE/PubMed, 3 titles in the Cochrane Central Register of Controlled Trials, 13 in the Web of Science and 20 in EMBASE. The first evaluation resulted in the selection of 11 complete articles. After careful reading, 5 studies [22–26] were excluded because they did not meet the eligibility criteria of this review (Table 2). Thus, 6 studies [27–32] published between 1997 to 2016 were included in the present systematic review. The search in the grey literature and clinicaltrials.gov database did not result in any further study. Fig 1 shows the process of searching and selecting articles. The k values of agreement between the two authors/reviewers for potential article inclusion (titles and abstracts) were 0.79 and 0.85 for the selected articles, which indicated excellent agreement [18].

**Table 2:** Excluded studies.

Reason for rejection	Authors
Case report	Binon (2005) [22]; Spinato et al. (2010) [23]; De Mendonça Invernici et al. (2014) [24]
Duplicate study	Krennmair et al. (2010) [25]
Review	Candel-Marti et al. (2011) [26]

### Study characteristics

The characteristics of the included studies are presented in Table 3. One cases series [27], one prospective cohort study [28] and four retrospective cohort studies [29–32] were included in the present systematic review. The number of participants ranged from 3 [27] to 205 [32], with a mean of 45.3 participants. Three hundred and fifty-seven implants were installed in subjects with primary or secondary SS. Two articles did not report the type of SS of the participants [29,30]. Prosthetic rehabilitations on the implants were conducted through unitary crowns [29–31], fixed partial dentures [29–31], complete fixed prostheses [27,31] or overdentures [27–31]. One study [32] reported only that the participants used fixed or removable prostheses, without discriminating prosthesis type. Only one study^31^ analyzed data from primary and secondary SS patients independently, with no significant difference for the periodontal parameters analyzed.

**Table 3:** Main characteristics of selected studies.

Authors (year)	Study desing Mean follow-up (months)	No. of subjects	Age Gender	Sjögren type (n)	Implants placed / implants failed	Mean survival rates (%)	Implant brand (surface) Implant Size (diameter x length) (mm)	No. of Smokers	Marginal Bone loss (mean ± SD) (mm)	Author’s conclusions
Payne et al. (1997)[27]	Case series 56	3	38–40 3F	Primary and secondary	26 / 3	88.4	Nobel Biocare (Machined) 3.75 x 10, 13, 18	1	NR	Despite some of the uncertainties of long-term prognosis, it is clear that benefits may be obtained from the placement of osseointegrated implants in selected SS patients.
Isidor et al. (1999)[28]	Prospective 48	8	53–70 8F	Secondary (NR)	54 / 7	87	Nobel Biocare (Machined) NR	NR	0.65 ± 0.07	Edentulous patients with Sjögren syndrome were most satisfied with the outcome of treatment when implant-retained fixed prostheses were used.
Weinländer et al. (2010)[29]	Retrospective 57.6	4	NR NR	NR	21 / 0	100	Camlog (Rough) 3.8, 4.3, 5.0 x 11, 13, 16	NR	3.1 ± 0.7	The clinical outcome of dental implant placement and implant prosthodontic rehabilitation was not negatively influenced in patients with autoimmune diseases such as rheumatoid arthritis or various types of connective tissue disease.
Öczakir et al. (2015)[30]	Retrospective 42	2	63–64 2F	NR	12 / 0	100	Straumann (Rough) NR	NR	NR	From the present report it can be seen that implant therapy is highly beneficial for patients with specific diseases and defects. Implants can be successful if these patients are given continuous professional support.
Korfage et al. (2016)[31]	Retrospective 46	50	67 46F / 4M	Primary (41) and Secondary (9)	140 / 4	97	NR NR	NR	0.89 ± 0.9	Based on the present analysis, we conclude that dental implants seem to be a favorable treatment option in the prosthetic treatment of patients with SS.
Albrecht et al. (2016)[32]	Retrospective 36	205	24–80 NR	Primary (156) and Secondary (49)	104 / 5	95.2	NR NR	76	NR	The high implant survival rate may encourage patients, rheumatologists, and dentists to consider dental implants for the treatment of patients with SS.

NR, not reported; SD, standard deviation; F, female

### Data synthesis

Regarding implant survival, an average of 94.6 ± 5.6% in a mean period of 3.97 years of follow-up was observed. No studies have reported whether implant failures occurred in patients with primary or secondary SS. In addition, the failures were also not correlated to the type of prosthetic rehabilitation.

Three studies [28,29,31] assessed the MBL level around the implants in a mean period of follow-up of 4.2 years. Radiographic assessment was performed through periapical [28] or panoramic radiographs [29,31]. The MBL outcomes are reported individually in Table 3.

Peri-implant parameters after implant installation were analyzed in two studies [29,31]. An article [29] observed a greater probing depth and gingival bleeding in SS patients when compared to healthy subjects. On another study [31], a higher number of mucositis and peri-implantitis in SS patients was observed, when compared to non-SS patients. Yet, there was no significant difference for the same parameters when compared with participants with primary SS or secondary SS.

No positive correlation between the evaluated peri-implant parameters and duration of SS or the use of immunosuppressive drugs was observed.

The masticatory function, oral comfort and satisfaction were evaluated in three studies [28,31,32] through completed questionnaires after rehabilitation with implants. All the articles observed positive results for the analyzed questions, showing an improvement in life quality of subjects with partial or total edentulous SS who were rehabilitated through dental implants.

### Assessments of the risk of bias and quality

Two studies [27,30] presented scores below 6 points, thus showing potential risk of bias. No article had the highest score (Table 4). No included study reported having followed a guideline to increase the research transparency (e.g. Strobe-Statement standards [33] for cohort studies).

**Table 4:** Assessment of quality and the risk of bias (NOS Scale).

Authors (year)	Selection				Comparability	Outcome			Total 9/9
	Representativeness of the exposed cohort	Selection of external control	Ascertaiment of exposure	Outcome of interest not present at start	Comparability of cohorts on the basis of the design or analysis	Assessment of outcome	Was follow-up long enough for outcomes occur	Adequacy of follow-up of cohorts	
Payne et al. (1997) [27]	0	0	★	0	★ 0	★	★	★	5/9
Isidor et al. (1999) [28]	0	0	★	★	★ 0	★	★	★	6/9
Weinländer et al. (2010) [29]	0	0	★	★	★ 0	★	★	★	6/9
Öczakir et al. (2015) [30]	0	0	★	0	★ 0	★	★	★	5/9
Korfage et al. (2016) [31]	★	★	★	★	★ 0	★	★	★	8/9
Albrecht et al. (2016) [32]	0	★	★	★	★ 0	★	★	★	7/9

## Discussion

### Summary of evidence

Patients with SS show a greater risk of developing cavities and early tooth loss because of an imbalance in salivary quality and flow [31,34–36]. Hence, rehabilitation through dental implants may return masticatory function, comfort, and esthetics to these Individuals. The aim of this systematic review was to investigate the implant survival rate, marginal bone loss and biological complications of dental implants in SS patients.

A comprehensive search for studies was carried out, including electronic search, manual search and gray literature. To reduce risk of bias, there was no restriction on language and publication dates.

Although RCTs are the studies with the least potential for bias [37], none can be included in this systematic review. From the six studies included in this systematic review, only one [28] was prospective.

Saliva, under normal conditions, has proteins, glycoproteins, enzymes, electrolytes and small organic molecules that promote lubricating, healing and antimicrobial action [38,39]. Hyposalivation, xerostomia or changes in saliva quality may compromise the teeth, but also bone integration or maintenance of peri-implant health [40].

No included study clearly correlated the reasons of implant failures. An analysis of the microbiological profile, cytokines, and biomolecular markers in sites with peri-implant disease of SS patients becomes essential in future research. Thus, defining the real influence of saliva on peri-implant health.

The chronic administration of corticosteroids commonly in patients with rheumatic diseases may induce an increase in osteoporosis levels, since there is a decrease in calcium intestinal absorption with simultaneous increase of renal excretion of this mineral [41,42]. Studies [43,44] in animals with osteoporosis have shown that healing and bone maturation may be delayed. Thus far, the impact of osteoporosis versus dental implants has not yet been well understood in the literature [45]. Two studies [31,32] included in this review reported on drug types administered by survey participants, yet no correlation between drugs versus implant failures was conducted.

Two recent systematic reviews [1,46] evaluating the survival rate of implants over 10 years in healthy patients, observed a mean survival rate of 96.5% and 95.3%, respectively. On the other hand, the present systematic review found 94.6 implant survival over a period of approximately 4 years of follow-up, which could indicate that there is a higher chance for implant loss in SS patients earlier in their treatment period.

A higher number of implant failures was verified in two included studies [27,28] when compared to others. The two papers, unexpectedly were the only ones that used machined surface implants. However, lack of information regarding the nature of failures (early/late) makes it impossible for conclusions to be drawn in regard to the influence of implant surface treatment and its impact on SS.

A recent systematic review [47], concluded that factors such as smoking, and periodontal disease may be more significant for the occurrence of long-term biological complications than surface treatment of implants. Two articles [27,32] included smoker participants. Despite evidence of the negative impact of smoking on implant survival [4,48], there was no clear correlation between the association of smoking and SS. The inclusion of smoking participants in the studies may bring a confounding factor for data interpretation.

According to Misch et al. [49], for success (excellent health), an implant should present an MBL<2 mm regardless of the follow-up period. Although only three studies [28,29,31] have analyzed MBL, good bone stability does not show differences in bone loss among individuals with or without SS. A justification for having a greater number of failures, and in contrast good stability in bone maintenance in the long term, is that most implant failures occur early, that is during the osseointegration process of the implants, as observed by a study [30] included in the present review.

Patients with secondary SS did not showed difference in implant rates when compared to primary SS [31]. Nevertheless, as patients with secondary SS are associated with another autoimmune disease, the influence of systemic disorders or additional medications should always be considered.

From the peri-implant parameters analyzed, mucositis was the most commonly reported. In fact, mucositis is the biological complication most commonly associated with dental implants [1,50], being characterized by inflammation of the soft tissues around the implants without signs of bone loss [51]. In one study [31], mucositis was observed in 94% of the SS carriers. Lower salivary flow, changes in salivary quality and immune compromising associated with SS may interact with factors commonly correlated with mucositis, such as poor hygiene and a narrow range of keratinized gingiva (<2 mm). In this way, patients with SS should be continuously motivated about hygienic methods and encouraged to follow a regular maintenance program.

### Strengths and limitations

This systematic review presents several strengths, such as a previous record of protocol, unrestricted search in the literature (including gray literature) selecting the best available evidence, searching process of studies, data extraction and risk analysis bias performed in duplicate.

Nonetheless, some limitations may be related to this systematic review. First, the low number of studies available in the literature with the absence of long-term prospective observational studies. Second, two included articles [27,30] presented a high risk of bias and their data should therefore be interpreted with caution. These studies did not report important data about the selection process of the patients (e.g. If the number of participants was representative in the community average).

In addition, no included study has determined the influence of "confusing factors" (e.g., smoking and medications) on study results.

### Recommendations for further research

As the current evidence is based on a low number of observational studies, researchers are encouraged to conduct a greater number of studies (preferably prospective) evaluating the performance of dental implants in SS patients. In addition, further investigation is needed on the influence of primary and secondary SS on implant results.

## Conclusions

With the limitations of this review and based on the available data, the dental implant therapy in SS patients seems to present high implant survival rate, low MBL and low biological complications. In addition, all included studies observed an increase in the quality of life of SS patients who were rehabilitated through dental implants. A greater number of prospective studies in the future is essential to support more robust conclusions.

## Supporting information

S1 Fig PRISMA checklist. (DOC)
